# Editorial Note: Protease nexin 1 induces apoptosis of prostate tumor cells through inhibition of X-chromosome-linked inhibitor of apoptosis protein

**DOI:** 10.18632/oncotarget.28901

**Published:** 2026-07-02

**Authors:** Chad M. McKee, Yunchuan Ding, Jianfeng Zhou, Chunrui Li, Liang Huang, Xiangke Xin, Jing He, Joshua E. Allen, Wafik S. El-Deiry, Yunhong Cao, Ruth J. Muschel, Danmei Xu

**Affiliations:** ^1^Department of Internal Medicine, Tongji Hospital, Tongji Medical College, Huazhong University of Science and Technology, Hubei, China; ^2^Gray Institute of Radiation Oncology and Biology, Medical Science Division, University of Oxford, Oxford, United Kingdom; ^3^Penn State Hershey Cancer Institute, Penn State University, Hershey, PA, USA; ^*^These authors have contributed equally to this work

The Oncotarget Scientific Integrity Office checked a data misplacement in the article: McKee CM, Ding Y, Zhou J, Li C, Huang L, Xin X, He J, Allen JE, El-Deiry WS, Cao Y, Muschel RJ, Xu D. Protease nexin 1 induces apoptosis of prostate tumor cells through inhibition of X-chromosome-linked inhibitor of apoptosis protein. Oncotarget. 2015 Feb 28;6(6):3784-96. doi: 10.18632/oncotarget.2921. PMID: 25686839; PMCID: PMC4414153.

Specifically, a duplication of the loading control beta-actin was found in Figure 4C (PC3 xenografts +/− pre-treatment with PN1) and Figure 5B (PC3 cells transfected with 2 μg Mock or PN1-expressing vectors for 24 h).

The Oncotarget Scientific Integrity Office contacted the authors to explain the data misplacement. The corresponding author Dr. DanMei Xu informed the journal that the authors revisited the archived materials, but the original raw western blot data for the actin loading controls are no longer available. They acknowledge that “the apparent duplication may have arisen from an error during figure assembly, but wish to point out that the equivalent loading of the lanes in Figure 5B is supported by an additional internal control (AKT) shown in the same panel. Furthermore, Figure 5D presents an extension of this experiment with the addition of an AKT inhibitor in which lanes 1 and 2 correspond to the same experimental conditions as those in Figure 5B and include appropriate actin controls. The conclusions of the study are supported by multiple independent experiments and complementary methodologies presented across our publications.”

As the identified issue cannot be corrected, in accordance with the COPE guidance* the journal is issuing an Editorial Note to inform our readers and maintain the transparency and integrity of the journal’s records.

*COPE guidance: “Concerns raised by a complainant that do not warrant a correction or expression of concern could also be resolved by some other means (e.g., letter to the editor, or note or notification).” https://publicationethics.org/guidance/guideline/expressions-concern.

Original article: Oncotarget. 2015; 6:3784–3796. 3784-3796. https://doi.org/10.18632/oncotarget.2921

